# Potential Predictive Value of Serum Pentraxin 3 and Paraoxonase 1 for Cardiometabolic Disorders Development in Patients with Psoriasis—Preliminary Data

**DOI:** 10.3390/metabo12070580

**Published:** 2022-06-22

**Authors:** Anna Baran, Anna Stepaniuk, Paulina Kiluk, Tomasz W. Kaminski, Magdalena Maciaszek, Iwona Flisiak

**Affiliations:** 1 Department of Dermatology and Venereology, Medical University of Bialystok, Zurawia 14 St., 15-540 Bialystok, Poland; anna.baran@umb.edu.pl (A.B.); paulina.kiluk@umb.edu.pl (P.K.); iwona.flisiak@umb.edu.pl (I.F.); 2 Pittsburgh Heart, Lung and Blood Vascular Medicine Institute, University of Pittsburgh, Pittsburgh, PA 15260, USA; kamins1@pitt.edu; 3 Department of Infectious Diseases and Hepatology, Medical University of Bialystok, Zurawia 14 St., 15-540 Bialystok, Poland; mm.maciaszek@wp.pl

**Keywords:** psoriasis, pentraxin 3, paraoxonase 1, comorbidities, cardiovascular biomarker, systemic therapy

## Abstract

Psoriasis is a systemic disease that is linked to cardiometabolic complications. Paraoxonase 1 (PON1) exerts anti-atherogenic properties. Pentraxin 3 (PTX3) is related to heart failure and atherosclerosis. We aimed to evaluate the protein levels in psoriatic patients and explore possible relations with disease activity, metaflammation parameters and systemic treatment. Thirty-three patients with plaque-type psoriasis and eleven healthy controls were enrolled in the study. Blood samples were collected before and after three months of therapy with acitretin or methotrexate. Serum proteins levels were evaluated using Bio-Plex 200 System. The mean serum pentraxin 3 level was significantly higher in patients with psoriasis, compared to controls (*p* < 0.01). Significant negative correlations between PTX3 with triglycerides in overweight patients, with glucose, cholesterol and triglycerides in obese patients, and with cholesterol and triglycerides in severe psoriatics were noted (all *p* < 0.05). After the treatment, PTX3 significantly decreased (*p* < 0.05). The mean serum PON1 in psoriatic patients did not differ, compared to the controls (*p* > 0.05). In psoriatics of normal weight, PON1 correlated negatively with liver enzymes activity (*p* < 0.05). PTX3 might exert a protective role in terms of cardiometabolic disorders development, especially in overweight and obese or most severe psoriatics. PON1 could serve as an indicator of the liver disorders in psoriasis.

## 1. Introduction

Psoriasis is characterized by erythematous-squamous plaques affecting mainly extensor surfaces but spreading to all body areas. Different clinical phenotypes of psoriasis have been reported, including palmoplantar, inverse, guttate, pustular, and others. Psoriasis may be associated with comorbidities, such as arthritis [1,2]. Even though psoriasis is considered to be a common disease, affecting 2–3% of the general population, its pathogenesis is not comprehended, but environmental, genetic, autoimmune and auto-inflammatory factors are considered to be potential causes [3,4]. It is believed that inflammatory cytokines, interleukin-1 and oxidative stress contribute to the development of the disease [5]. Moreover, psoriasis is linked to systemic metabolic disorders, including obesity, hypertension, metabolic syndrome (MS), dyslipidemia and diabetes mellitus (DM) [5,6]. It has been widely proved that cardiovascular complications lead to a reduction in life expectancy among patients with psoriasis which additionally adds pressure to explain its pathogenesis and develop novel markers to assess the potential risk of comorbidities development. We have been constantly exploring various active proteins in psoriatic patients and demonstrated them as novel indicators of inflammation or the development of metabolic complications [7,8]. 

Human serum paraoxonase 1 (PON1) is a 43 kDa glycoprotein that is composed of 354 amino acids, synthesized in the liver and secreted to the bloodstream and associated with the plasma high-density lipoprotein (HDL) of activities of both paraoxonase (PON) and arylesterase (ARE) [9,10]. PON1 is calcium-dependent and believed to prevent the molecules’ oxidation, the peroxidative damage of the cell membranes, and organophosphorus exposure [5,9]. Moreover, PON1 protects low density proteins (LDL) from oxidation and is therefore believed to act against the development of coronary heart disease which is reported in a higher prevalence among patients with psoriasis [5,11,12]. Beside its beneficial antioxidant properties, an antiatherogenic role of PON1 has been widely proposed [11]. The protein influences glucose tolerance and insulin sensitivity by upregulating the expression of the glucose transporter 4 (GLUT4) [11]. Given the strong connection between psoriasis and DM, impaired glucose tolerance can significantly worsen the course of other diseases that are related to MS and psoriasis as well. Non-alcoholic fatty liver disease (NAFLD) is another disorder that is associated with both psoriasis and DM as the liver plays an important role in glucose uptake and impairs insulin secretion [11]. By protecting from inflammation and oxidative stress, PON1 is believed to take part in the pathogenesis not only of NAFLD, but also cardiovascular or neurological disorders, which are all closely related to psoriasis [11,13]. Decreased PON1 was reported among patients with a diet that was rich in high fat, and obese patients, since obesity has multidirectional interrelations with psoriasis [11]. The data on serum PON1 activity in psoriasis are divergent. Ferretti et al. reported lower activity among patients with psoriasis, compared to controls [14]. Similarly, Prathibha et al. showed that serum paraoxonase 1 level was significantly decreased in psoriasis patients, compared to controls [12]. In contrast, Toker et al. reported significantly higher serum PON1 activity in patients with psoriasis, compared to the healthy group [15]. Taking this into consideration, PON1 seems to be a very promising biomarker for the detection of cardiometabolic complications in psoriatic patients. However, divergent data and the interindividual variabilities of PON1 still challenge the growing knowledge.

Pentraxin 3 (PTX3) is an acute-phase reactant, produced in reaction to the stimulus of the inflammatory reaction that is mediated by TNFα and IL-1, among others [3,6,16]. PTX3 is a member of the pentraxin superfamily and its structure and function is similar to CRP (C-reactive protein) [3,16]. PTX3 can be found in neutrophiles, macrophages, fibroblasts, adipose cells and vascular endothelial cells [16]. PTX3 has been linked with heart failure and atherosclerosis, and elevated levels were found among patients with coronary artery disease (CAD) [17]. The protein has been proposed as an early indicator of myocardial infarction and a prognostic factor of mortality rate in CVD (cardiovascular disease) [18]. PTX3 was also increased in chronic kidney disease (CKD) as a result of the inflammatory process starting at the early stages, which is also interrelated to psoriasis [16,19,20]. Furthermore, higher PTX3 levels were reported among patients with severe psoriasis, compared to those with mild psoriasis or healthy individuals [3]. However, the data on PTX3 and psoriasis are ambiguous as in another study the levels remained unchanged in patients with psoriatic arthritis [6]. Therefore, further research should be encouraged.

Considering the promising role of pentraxin 3 and paraoxonase 1 in atherosclerosis-related diseases and the convergent data that have been reported, we aimed to investigate the serum levels of these proteins in psoriatic patients, in order to explore their potential diagnostic usage in this disease. We evaluated the relations of the proteins with psoriasis severity, metabolic and inflammatory indicators, as well as in relation to systemic therapy. 

## 2. Results

The study included 33 patients with exacerbated plaque-type psoriasis, 15 women and 18 men, with the mean age of 54.2 ± 16.8, and 11 healthy individuals of the mean age of 54.4 ± 9.11, matched for age, weight, and BMI (Body Mass Index). The severity of psoriasis expressed by PASI (Psoriasis Area and Severity Index) score was 17.12 ± 7.23 before treatment and 4.22 ± 2.88 after therapy (*p* < 0.001). The characteristics of the study groups are summarized in Table 1 and Table 2. Additional, more accurate analysis of the patients with regard to BMI and PASI subdivision, along with particular drug subgroups, have been placed in the Supplementary Materials (Tables S1 and S2).

PASI—psoriasis area and severity index; RBC—red blood cells; PLT—platelets; WBC—white blood cells; TGs—triglycerides; HDL—high-density lipoproteins; LDL—low-density lipoproteins; CRP—C-reactive protein; ALT—alanine transaminase; ASPAT—asparagine transaminase.

### 2.1. Pentraxin 3 (PTX3)

**Figure 1:**
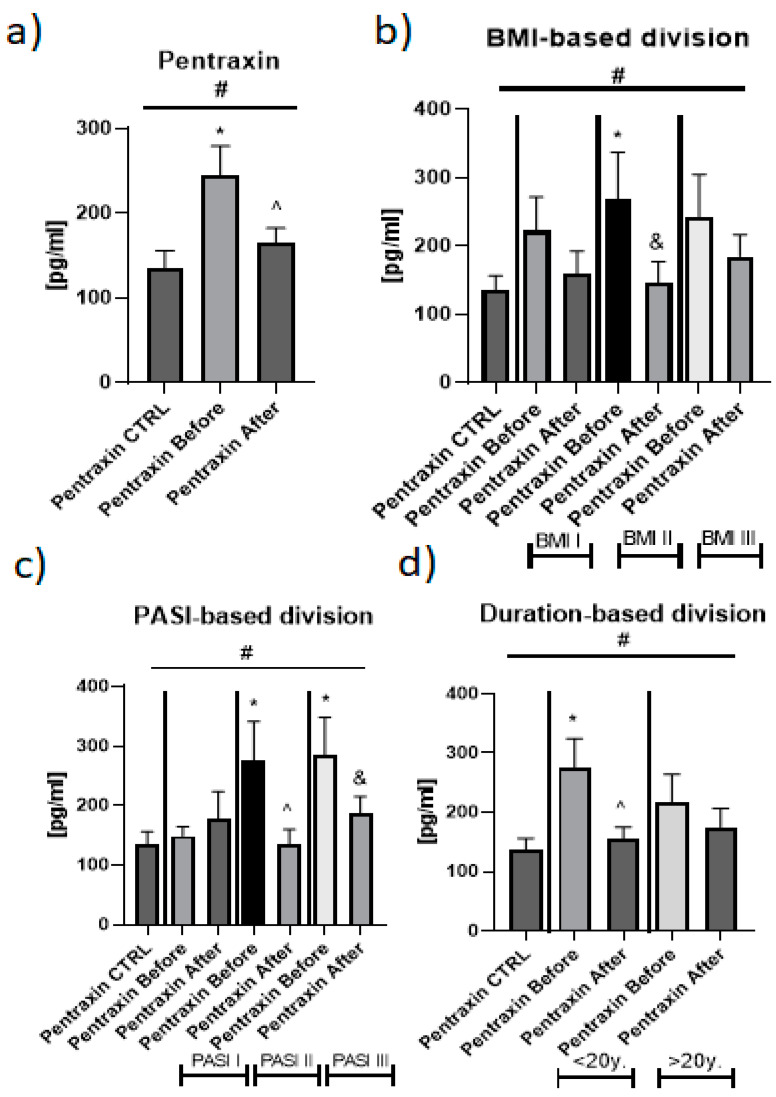
The levels of PTX3 in psoriatics before and after total treatment (**a**) and divided into BMI (**b**), PASI (**c**) and disease duration (**d**) subgroups in comparison to the controls. *—means the existence of statistically significant difference between patients single group, compared to controls with *p* < 0.05. and &/^—means the significance when comparing the levels before and after treatment inside the subgroups. #—shows the statistical significance between controls and marked patients’ subgroups when compared using ANOVA with *p* < 0.05.

The mean serum pentraxin 3 concentration in patients with psoriasis was 245 ± 34.2 pg/mL before treatment and 165 ± 18.1 pg/mL after, and it was significantly higher compared to the controls, who had a concentration of 136 ± 20.5 ng/mL (*p* < 0.01) (Figure 1a). 

Regarding the BMI subdivision, pentraxin 3 level was the highest in overweight patients with significance, compared to the healthy individuals (Figure 1b). Further, in PASI II and PASI III serum, PTX3 concentration was significantly elevated, compared to the controls (Figure 1c). In turn, duration-based division with the threshold of 20 years revealed that in patients with short-term psoriasis (<20 years), the pentraxin 3 level was significantly higher than in those with long-term disease (>20 years) (Figure 1d). The ANOVA test showed significant relations between the controls and subgroups of patients that were subdivided with accordance to BMI, PASI, duration or particular drug (Figure 1).

Regarding the relations of the protein and laboratory parameters, pentraxin 3 significantly positively correlated with CRP (and such a trend was observed in relation with PASI—Table S4, Supplementary Materials). Further, in patients of normal weight, PTX3 was related positively with PASI, WBC and CRP at statistical significance (Table S5a, Supplementary Materials). In overweight negative correlations between the protein and RBC, HGB, WBC and TG were noted (Table S5b, Supplementary Materials). In obese psoriatics PTX3 correlated positively significantly with PASI and CRP and negatively with RBC, glucose, cholesterol and triglycerides levels (Table S5c, Supplementary Materials). Regarding PASI-based division, in patients with mild psoriasis, significant negative correlations between pentraxin 3 and PASI or PLT, and positive correlations with HGB were noted (Table S6a, Supplementary Materials). In moderate-to-severe psoriatics, PTX3 was negatively related with HGB, RBC and AST activity while it was positively correlated with glucose level (Table S6b, Supplementary Materials). In severe psoriasis, significant negative correlations of pentraxin 3 level and RBC, Chol and TG were observed (Table S6c, Supplementary Materials).

After the therapy, a clinical improvement was achieved that was expressed through a significant decrease in PASI score (*p* < 0.01) (Table 2). The 3-month treatment resulted in a significant decrease in serum PTX3 concentration, which did, however, stay significantly higher than in the controls (*p* < 0.05) (Figure 1a).

**Figure 2:**
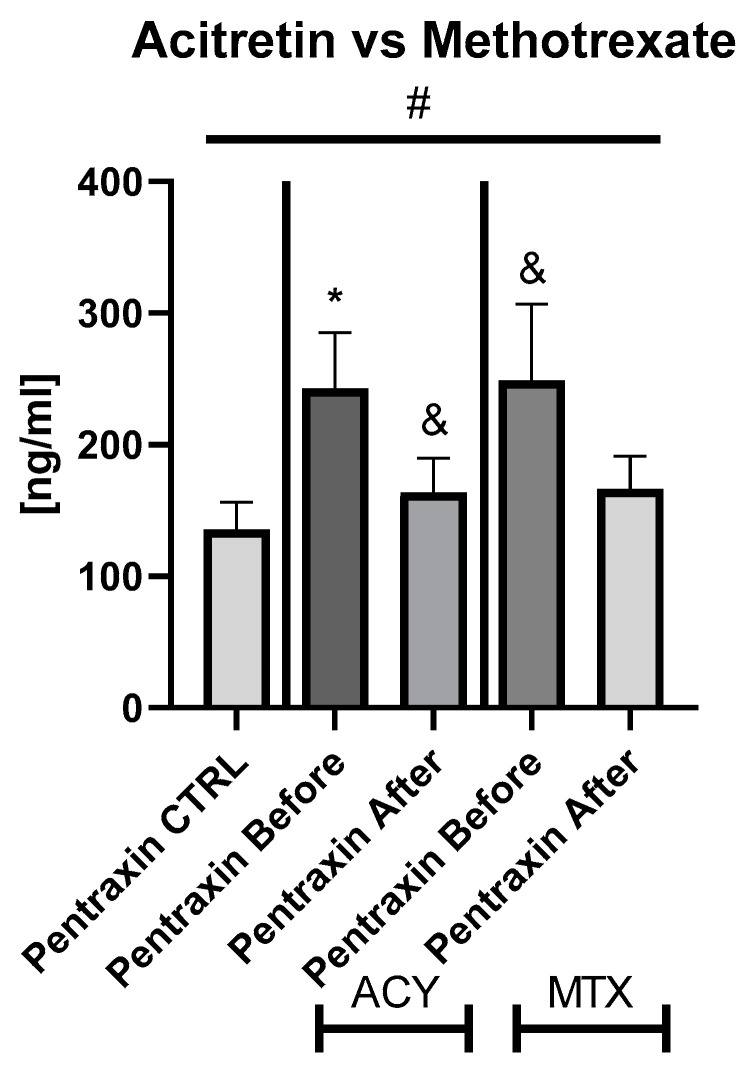
The levels of PTX3 in psoriatics divided into subgroups undergoing therapy separately with acitretin and methotrexate, compared to controls. *—means the existence of statistically significant difference between patients single group, compared to controls with *p* < 0.05. and &—means trend—*p* < 0.1. #—shows the statistical significance between controls and marked patients’ subgroups when compared using ANOVA with *p* < 0.05.

Regarding the impact of particular drug on PTX3 level, both MTX and acitretin caused a decrease in the protein level, however, therapy with the latter acted significantly (*p* < 0.05) (Figure 2).

As for the duration-based division, significance was observed in short-term psoriasis in which the total treatment resulted in a meaningful decrease in PTX3 concentration (*p* < 0.05) (Figure 1d). Similarly, in overweight patients, pentraxin 3 level significantly decreased after the therapy (*p* < 0.05) (Figure 1b). PASI-based division showed a significant decrease in PTX3 concentration in moderate-to-severe and severe psoriasis (Figure 1c).

With reference to the relations between pentraxin 3 level and laboratory parameters after total treatment, only a significant negative correlation with Chol was noted (Table S4b, Supplementary Materials). When looking at the particular drug influence, after therapy with acitretin, a significant positive relation with PASI was maintained together with the occurrence of negative correlations with WBC, glucose and Chol (Table S7a, Supplementary Materials). In patients that were treated with MTX primary, a positive link with PASI disappeared, but a positive one was found with WBC (Table S7b, Supplementary Materials). As for selected relationships inside the BMI subgroups after therapy, in patients of normal weight, a significant positive correlation of PTX3 with liver enzymes activity was noted (Table S5a, Supplementary Materials). In overweight psoriatics, the protein was negatively related with WBC, PLT, glucose and cholesterol levels (Table S5b, Supplementary Materials). In obese patients, PTX3 was linked statistically positively with ALT activity and negatively with Chol ((Table S5c, Supplementary Materials). Regarding selected significant relations inside PASI subgroups after therapy, in PASI I, pentraxin 3 level was positively correlated with WBC, PLT, glucose and CRP levels (Table S6a, Supplementary Materials). Further, in PASI II subgroup, negative correlations of PTX3 with PASI and Chol level were observed (Table S6b, Supplementary Materials). In PASI II, the protein correlated negatively with PASI and cholesterol level at statistical significance. In most diseased patients, PTX3 was linked significantly negatively with WBC, glucose and Chol and positively with liver enzymes activity (Table S6c, Supplementary Materials).

### 2.2. Paraoxonase 1 (PON1) 

**Figure 3:**
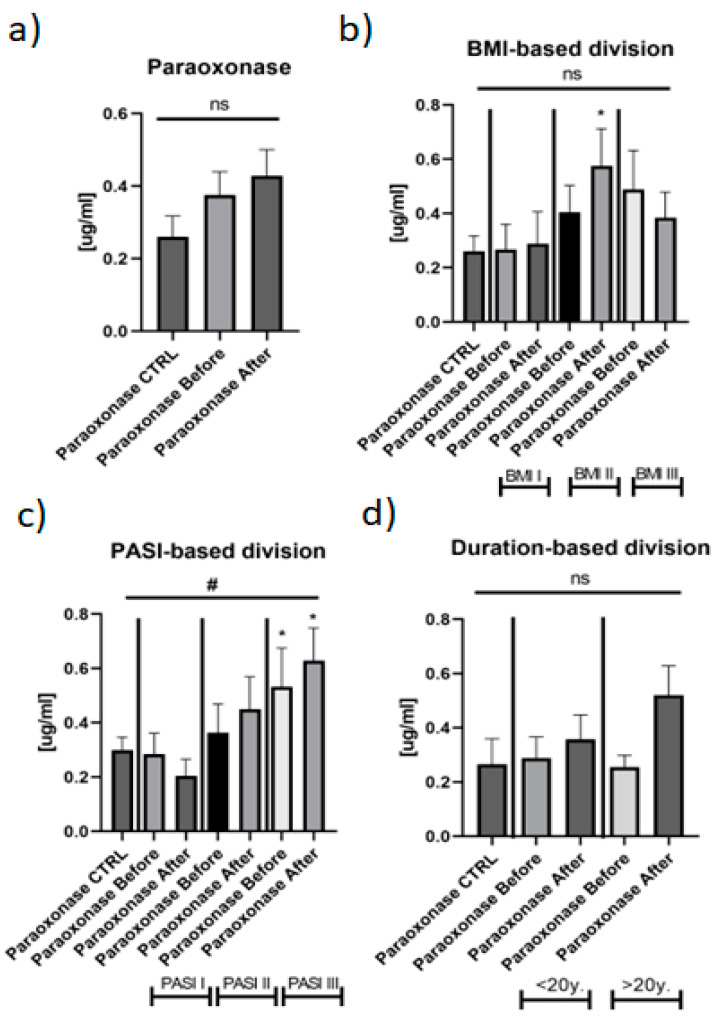
The levels of PON1 in psoriatics before and after total treatment (**a**) and divided into BMI (**b**) and PASI (**c**) subgroups in comparison to the controls. *—means the existence of statistically significant difference between patients single group, compared to controls with *p* < 0.05; (**d**) The duration-based division did not reveal any meaningful relations in the protein level and with reference to the treatment. ns, non-significant. #—shows the statistical significance between controls and marked patients’ subgroups when compared using ANOVA with *p* < 0.05.

The mean serum paraoxonase 1 concentration in patients with psoriasis was 0.404 ± 0.068 ug/mL before treatment and after 0.430 ± 0.069 ug/mL, and this did not differ significantly, compared to the controls: 0.228 ± 0.047 ug/mL (*p* > 0.05) (Figure 3a). 

Regarding BMI-based division, no important differences were noted between the subgroups and controls in paraoxonase 1 levels (Figure 3b). In most severe patients, PON1 was the highest and most statistically elevated in comparison with the healthy persons and the other PASI subgroups (Figure 3c). The duration-based division did not reveal any meaningful relations in the protein level and with reference to the treatment (Figure 3d). 

Regarding the relations of PON1 with laboratory parameters, no important correlations were noted (Table S4, Supplementary Materials). However, in psoriatics of normal weight, paraoxonase 1 correlated negatively with liver enzymes activity (Table S5a, Supplementary Materials); in overweight patients, PON1 correlated positively with glucose level and negatively with AST activity. Finally, in obese patients, PON1 was related negatively with PLT, glucose and triglycerides levels (Table S5c, Supplementary Materials). According to PASI subgroups, in patients with mild psoriasis, PON1 significantly positively correlated with WBC, CRP, PLT, Chol and AST activity (Table S6a, Supplementary Materials). No such interrelations were noted in the PASI II subgroup. In contrast, in patients with severe psoriasis, significant negative correlations of PON1 with CRP and AST activity were observed (Table S6c, Supplementary Materials).

**Figure 4:**
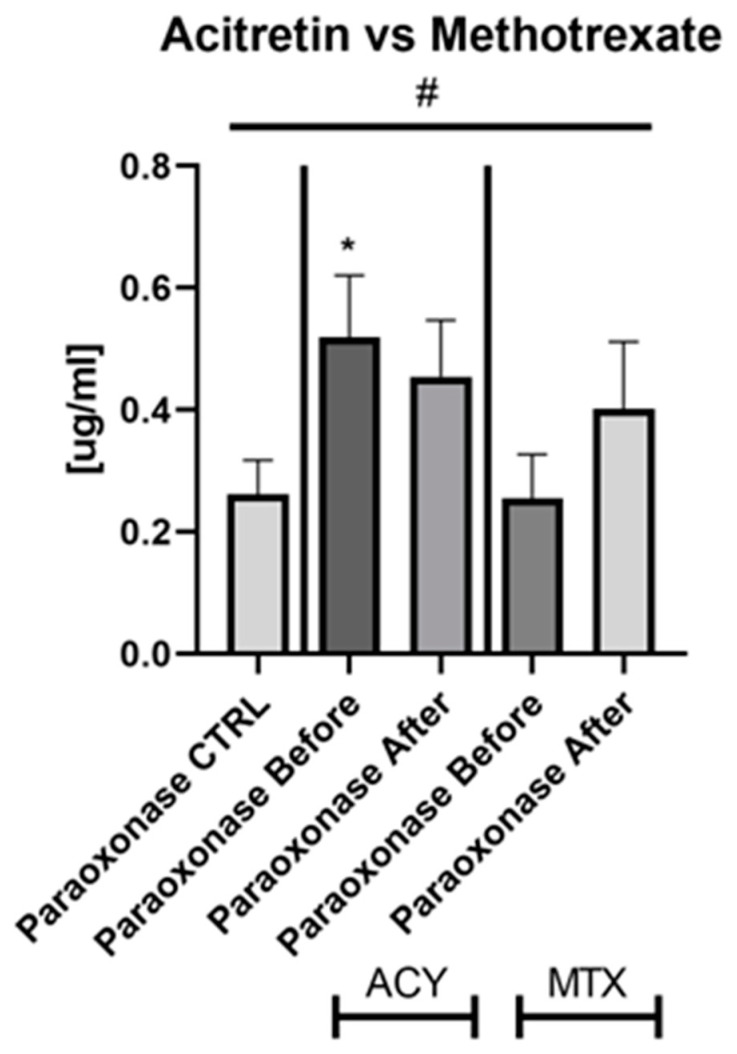
The levels of PON1 in psoriatics divided into subgroups undergoing therapy separately with acitretin and methotrexate, compared to controls. *—means the existence of statistically significant difference between patients single group, compared to controls with *p* < 0.05. #—shows the statistical significance between controls and marked patients’ subgroups when compared using ANOVA with *p* < 0.05.

Serum paraoxonase 1 level did not change significantly after treatment with both drugs in total and separately, however, after MTX it increased insignificantly (Figure 3a and Figure 4). 

In overweight patients, PON1 increased significantly after total therapy and was significantly higher than in the controls (Figure 3b). In the PASI III subgroup, the protein stayed elevated and remained the highest when compared to the other subgroups and healthy subjects (Figure 3c). Regarding relations with laboratory parameters, PON1 correlated positively with CRP level after treatment (Table S4b, Supplementary Materials). In overweight psoriatics, significant negative correlations between paraoxonase 1 and HGB, glucose, Chol and TG levels were noted after therapy (Table S5b, Supplementary Materials). Further, in obese patients, PON1 was significantly positively related with PASI and negatively with HGB, RBC and glucose levels (Table S5c, Supplementary Materials). Regarding particular drug impact, significant positive correlations between PON1 and PASI and CRP were noted after acitretin, but not MTX (Table S7, Supplementary Materials). After, methotrexate paraoxonase 1 correlated negatively with glucose level (Table S7b, Supplementary Materials).

## 3. Discussion

Psoriasis is a very complex systemic disease that is linked to an increased risk of developing other chronic conditions, including hypertension, dyslipidemia and glucose intolerance, among others. The wide range of comorbidities highlight the urgent need to develop predictors allowing to adequately assess patients’ risk, implement early prophylaxis and proper effective treatment that is adjusted to the clinical status, and result in an increase in the quality of life and life expectancy. Therefore, we aimed to elucidate the potency of PON1 and PTX3, as they could be used as novel regulators of cardiometabolic disorders in psoriasis in terms of systemic therapy.

In our study, serum pentraxin 3 level was significantly higher in patients with psoriasis, compared to the healthy subjects. Our results stay in line with other studies which also demonstrated elevated PTX3 levels in psoriatic patients [3,6,21,22]. Beveluacqua et al. noted an increased production of the protein, not only in plasma, but also in the supernatant of purified monocytes from patients with severe psoriasis [22]. The authors suggested the significant relation between cellular production and plasma levels of PTX3 as a sign of cellular activation by monocytes/macrophages that first infiltrates the psoriatic lesion. Further, a strong PTX3 staining in fibroblasts, endothelial cells and monocytes/macrophages in the lesional skin of severe psoriatics was detected [22]. These findings, along with ours reflect a certain role of PTX3 in psoriasis pathogenesis. Furthermore, we noted a significantly increased PTX3 level in overweight psoriatics and interesting various relations with metabolic and inflammation indicators levels in certain study subgroups—especially overweight and obese ones. These outcomes point to the uncertain role of pentraxin 3 in the interrelations between inflammation and adipose tissue in psoriasis. Gathering our own results and the available data, we could assume that PTX3 might be a novel early indicator and a protector of cardiometabolic disorders in psoriatics (especially overweight and obese), and furthermore, with a severe form of the disease and in the short-term. We observed a positive trend in relation to PTX3 with PASI, and a significant positive correlation in psoriatics of normal weight and obesity. Further, the protein level was significantly higher in moderate-to-severe and severe psoriatics than in the controls. To highlight more these interrelations, in one study, among overweight and obese children, the level of PTX3 was significantly higher than in the controls of normal weight and corresponded with the thickness of both epicardial adipose tissue and carotid intima media [23]. This finding suggests the potential role of the molecule as a marker of the cardiovascular risk, which is even more crucial among patients with psoriasis, as the disease is linked to the greater carotid intima-media thickness and overproduction of pro-inflammatory adipokines by the epicardial adipose tissue, which in turn also influences PTX3 levels [23,24,25]. Comparing with the other authors’ results, Bevelacqua et al. reported a statistical positive correlation between PTX3 and PASI score and also suggested that the protein could be used to measure the disease’s activity [22]. In our study, PTX3 was positively correlated with CRP level which reflects that the protein acts as an indicator of inflammation in psoriasis. Similarly, a prognostic value of PTX3 has also been demonstrated in myocardial infarction or CAD [17]. Gathering our own and cited results, along with elevated levels in MS, CVD, obesity or DM, we can conclude that PTX3 could be used as a novel marker of metaflammation and atherosclerosis-related disorders in psoriasis. Interestingly, significant negative correlations between PTX3 with triglycerides in overweight patients; with glucose, cholesterol and TG in obese; and with Chol and TG in severe psoriatics might suggest a protective role of the protein in cardiometabolic disorders (CMDs) development, especially within intensifying metaflammation in psoriasis. In a study conducted by Sabry et al. among 75 patients with psoriasis, both LDL and serum PTX3 were significantly higher, whereas serum HDL was significantly lower, compared to the control group [26]. Qin et al. observed the same correlation among elderly patients with acute cerebral infarction and reported a positive correlation between PTX3 and lipids levels, as well as inflammatory markers [27]. Interestingly, statistically higher PTX3 levels in short-term psoriasis might reflect a greater protective impact of PTX3 in these patients, and perhaps in long-lasting psoriasis, other pro-inflammatory stimuli may enhance the ongoing metaflammation.

After the total systemic therapy, we observed a significant decrease in pentraxin 3 serum level, which stayed higher than in the healthy subjects. Regarding the particular drug’s effect, acitretin was the one which resulted in the significant decrease in PTX3 level. Presumably, the interaction of classical anti-psoriatic therapy with the protein has a beneficial—but not yet sufficiently effective— cardioprotective effect. Similarly, Ctirad et al. observed a significant decrease in PTX3 level after combined therapy with UV light and topical coal tar [21]. In a study by Deyab et al., after 6 months of anti-rheumatic treatment with methotrexate or anti-tumor necrosis factor alpha with and without methotrexate among patients with inflammatory rheumatic diseases, PTX3 did not improve as the only one of the inflammatory markers [28]. Furthermore, neither of the administered drugs resulted in the reduction in PTX3 levels in that study [28]. Therefore, Deyab et al. suggested that PTX3 may be a marker of the continuous inflammatory process that cannot be suppressed by the standard treatment [28]. Yaman et al. examined the effect of acetaminophen overdose on PTX3 levels and reported its elevated level in rats. The obtained results demonstrated that the protein could be potentially used as an indicator of acute liver damage [29].

Sparse data regarding the impact of anti-psoriatic treatment on pentraxin 3 levels together with the data that were obtained by us make it impossible to draw unequivocal conclusions, but should encourage further exploration.

Numerous studies reported lower paraoxonase 1 activity in patients with obesity or past myocardial infarction, suggesting that the low activity of PON1 serves as a CVD risk factor [11,18]. It was also observed that decreased PON1 activity was related to the increased occurrence of atherogenic dyslipidemia [30]. Bednarz-Misa et al. noted a significantly decreased PON1 level among patients that were admitted to the intensive care unit with multiple organ disfunction syndrome (MODS) and reported that lower PON1 activity was associated with cardiovascular insufficiency [31]. Moreover, Bednarz-Misa et al. suggested that PON1 could be used as a marker of cardiovascular insufficiency with an accuracy of 82% [31]. It is known that inflammation and oxidative stress are distinctive features of sepsis and MODS, which is a complication of the former [31]. This report highlights that PON1 can be a marker of the increased oxidative damage [11]. The available data on PON1 in psoriasis are conflicting. In the previous studies, its activity was reported to be lower than in the controls [12,18]. Therefore, patients with psoriasis were suggested to be considered at higher risk for atherosclerosis development [12]. On the other hand, Ferretti et al. noted that serum PON1 was lower in psoriasis subjects with regard to controls, however this was without significance [18]. Khoshnoodi et al. found no significant differences in PON1 activity between psoriatics and controls and no relationships with dyslipidemia indicators [32]. We also did not observe any important differences in the serum level of PON1 between psoriatics and controls regarding BMI-based division. However, surprisingly, in severe psoriatics serum PON1 level was the highest and significantly higher than in the healthy subjects. Noteworthy, Toker et al. noted significantly elevated PON1 concentration in psoriatics, compared to the healthy individuals [15]. 

Sorokin et al. also reported elevated levels of this molecule among patients with psoriasis, in contrast to the control group [33]. The researchers explained the increase as a way to compensate the escalated oxidation processes [33]. Furthermore, they observed higher levels of oxHDL and oxLDL among patients with psoriasis [33]. HDL is known to prevent from coronary disease, but due to oxidation HDL loses its properties and can promote vascular calcification which leads to a pro-atherosclerotic effect [34,35]. Sorokin et al. mentioned that worse prognosis in terms of cardiovascular disease was linked to lower PON1 levels [33]. A possible explanation of this phenomenon is that PON1 has a role in the process of preventing LDL from oxidation by HDL molecules and eliminating already oxidized lipids [36]. Interestingly, Asefi et al. demonstrated that the presence of the PON1 55 M allele was associated with psoriasis (odds ratio = 1.96, *p* = 0.017) [37]. The authors indicated that oxidative stress, impairment of the antioxidant system and abnormal lipid metabolism play a role in the pathogenesis and progression of psoriasis, and pointed out that patients with psoriasis are more susceptible to vascular diseases [37].

These divergent results cited along with ours highlight the potential protective role of paraoxonase 1 in terms of the intense metaflammation in severe psoriasis. In fact, various modifiers, such as methodological nuances, genetic and environmental or individual factors, such as lifestyle or alcohol consumption have been proved to influence the different results and activities of PON1 [18]. Worth highlighting are the negative correlations between the protein and liver enzymes activity in certain PASI subgroups. Presumably, PON1 might be an indicator of the liver dysfunction in psoriasis. Further studies are definitely needed to explore this issue.

Data regarding the impact of anti-psoriatic systemic treatment on paraoxonase 1 are very little. We did not note any important influence of therapy on PON1 level, however, MTX resulted in an insignificant increase in the protein level. These could point to an insufficient additive role of systemic therapy in minimizing atherosclerosis risk in psoriasis. However, methotrexate seems to be more beneficial. Pektas et al. did not demonstrate any meaningful impact of UVB-NB on the serum PON1 level in patients with psoriasis [18]. In the study of Kilic et al., just as in ours, there was no significant variation in paraoxonase levels pre- and post-treatment with 8 weeks of methotrexate therapy [38]. Bacchetti et al. noted a significant increase in PON1 activity and an increase in the PON1/CRP ratio after 24 weeks of treatment with etanercept in patients with psoriasis [39]. The fact that CRP and PON1 activity have a substantial inverse association shows that PON1 activity and inflammation are linked in psoriasis. The results pointed out that treatment with etanercept is correlated with a reduction in lipid peroxidation and an improvement in HDL antioxidant and anti-inflammatory properties [39].

In one study, it was reported that among patients with rheumatoid arthritis who were treated with methotrexate, their HDL function profile improved after treatment [40]. It was also noted that patients that were treated with tocilizumab due to an inadequate response to methotrexate had lower levels of HDL-associated SAA [40]. PON1 in this group significantly increased after the treatment and a decrease in CRP level was observed which was attributed to a reduced inflammatory process [40]. In our research PON1 increased after treatment with MTX. However, we observed that after treatment, PON1 correlated positively with CRP.

There is little research on the impact of certain systemic therapeutic methods on the evaluated proteins levels, especially in psoriasis. However, considering the limitations of our study, a small number of participants (especially in the subgroups) or a single process of protein detection could have altered the results which should be treated as preliminary, not conclusive, but still promising. Unfortunately, the pandemic prevented us from reaching a larger study group. To fully rule out the bias results, further research will be conducted on a larger number of subjects, perhaps with different systemic methods of treatment, including biologicals. Therefore, our outcomes should be regarded as exploratory and encouraging rather than final. A primary limitation of our study was a relatively small number of individuals participating in the research. The patients were a representation that were specific for Podlaskie Voivodship, which is not entirely representative. Therefore, the obtained results may not be accurate for the general population. The limited number of participants in the control group could also potentially alter the findings. 

## 4. Materials and Methods

This prospective study involved 33 adult patients (18 male and 15 female) with exacerbation of plaque-type psoriasis. Eleven healthy individuals, matched for sex, age and BMI were used as a reference for the serum levels of pentraxin 3 and paraoxonase-1 and selected laboratory parameters. We excluded from the study patients who were pregnant, breastfeeding, those who had undergone anti-psoriatic treatment within the last four weeks, as well as those with hypertension, diabetes mellitus, chronic renal or heart failure, liver disease, acute or chronic infection, other autoimmune diseases and malignancy. Psoriasis area and severity index (PASI) was evaluated by the same investigator in all patients at the time of the visit before and after the therapy introduction. The patients were stratified depending on their PASI into 3 subgroups: mild (PASI 1), meaning a score under 10 points, noted in 8 patients; moderate (PASI 2), a score of 10–20 points noted in 13 persons; and severe psoriasis (PASI 3), related to PASI > 20 points and calculated in 12 persons. The patients were also divided regarding BMI (Body Mass Index, calculated as the body weight of participants divided by the square of height); group 0 meant the healthy group, BMI 1—ten normal-weight psoriatics (18.5–24.9); BMI 2—eleven overweight subjects (BMI 25–29.9); BMI 3 was for obesity (BMI > 30) and was noted in 11 patients. Levels of highly sensitive C-reactive protein (hs-CRP); complete blood count (CBC); serum glucose; total cholesterol (Total Chol); HDL (high-density lipoprotein); LDL (low-density lipoprotein); triglycerides (TG); and indicators of kidney and liver functions were evaluated in the study and control group before and after therapy in psoriatics. Blood samples were collected before and repeated after three months of systemic treatment with 15 mg/week of methotrexate (MTX) (19 patients), or acitretin (ACY) (14 persons) in a bodyweight dose of 0.5 mg/kg/day, considering patients’ general internal condition, tolerability and indications. Written informed consent was obtained from all the subjects. The study was approved by the local Bioethical Committee (Protocol number R-I-002/354/2015) and was in accordance with the principle of the Helsinki Declaration.

### 4.1. Serum Collection and Evaluation 

Fasting blood samples were taken from all the study and control groups using vacutainer tubes and allowed to clot for 30 min. The samples were centrifuged for 15 min at 2000× *g* and then the separated serum was frozen immediately for storage at −80 °C and further analysis. The blood samples for biochemical tests and blood counts were collected at the same time as other tubes and performed by routine laboratory techniques using an automated analyzer, Cobas 6000 c501 (Roche Diagnostics, Rotkreuz, Switzerland) and a Sysmex XN-1000 hematology analyzer. The serum level of assay parameters was measured using a validated and calibrated Bio-Plex 200 System, according to the manufacturer’s instructions, provided by Bio-Rad. After 3-months of the continuous systemic therapy, blood samples were taken from the patients, and the protein levels and laboratory parameters were re-evaluated. At the same time points, BMI and psoriasis severity by the PASI Index were assessed.

### 4.2. Statistical Analysis 

The normality of the obtained results in terms of distribution was tested using the Shapiro–Wilk test and quantitative data were expressed as mean  ±  SD. The non-Gaussian data were shown as a median (with the full range values). The Student’s *t*-test or nonparametric Mann–Whitney test were used to compare the differences between the studied group and healthy individuals; for binary data a Chi-square test was performed. The analysis of variance or Kruskal–Wallis test was used to assess the differences between the study subgroups and this was followed by a Bonferroni post-hoc analysis when appropriate. The correlations between the variables were calculated using a Spearman’s rank correlation analysis. A two-tailed *p* value of <0.05 was considered to be statistically significant. Computations were performed using GraphPad 9 Prism Software (GraphPad Software, La Jolla, CA, USA). The power of the analysis was estimated using StatMate 2 Software (GraphPad Software, La Jolla, CA, USA). 

## 5. Conclusions

Our findings highlight the significant role of PTX3 and PON1 in chronic inflammation and psoriasis severity. PTX3 may serve as a protective protein regarding the development of cardiometabolic disorders, especially in overweight and obese patients with psoriasis. Moreover, we noted a statistically higher PTX3 level among patients with short-term psoriasis which suggests greater protection from oxidation among this group. The highest level of PON1 among patients with the most severe psoriasis leads to the conclusion that the increase may be a compensation of the advanced inflammatory process. Considering the negative correlations between paraoxonase 1 and liver enzymes activity, it can be assumed that PON1 might be a novel indicator of the liver dysfunction in psoriasis; however, further research is needed. The significant increase in PON1 among overweight patients after treatment suggests that this group benefits the most in terms of the reduction in oxidation and inflammation. Furthermore, anti-psoriatic systemic therapy in relation to the evaluated proteins does not seem to be sufficiently cardioprotective; methotrexate seems to be more beneficial.

## Figures and Tables

**Table 1:** Baseline characteristics of patients and controls.

Parameter	Controls (*n* = 11)	Psoriasis Patients (*n* = 33)
Age	54.4 ± 9.11	54.2 ± 16.82
Sex (M/F)	4/7	21/12
Height	166 ± 8.83	171 ± 10
Weight	69.4 ± 15.21	74.8 ± 20.90
BMI	25 ± 3.59	26.9 ± 6.42

**Table 2:** Basal characteristic of the patients’ group before and after total treatment.

Parameter	Before Treatment	After Treatment
PASI	17.12 ± 7.23	4.22 ± 2.88 ***
HGB [g/dL]	13.55 ± 1.72	13.21 ± 1.48
RBC [×10^3^/mL]	4.37 ± 0.57	4.29 ± 0.48
WBC [×10^3^/mL]	7.65 ± 1.88	6.63 ± 1.61 *
PLT [×10^3^/mL]	255 ± 74.29	231.70 ± 58.99
Glucose [mg/dL]	85 (53–215)	91.73 ± 13.55
Hs-CRP [mg/L]	5.30 (1–36.20)	3.44 (0.50–13.50) *
ALT [U/L]	24.89 ± 11	21.09 ± 8.55
AST [U/L]	23.90 ± 12.55	21.67 ± 1.22
Total Chol [mg/dL]	173.60 ± 40.13	171.4 ± 39.10
TG [mg/dL]	136 ± 63.70	120.8 ± 56.68

*/*** means statistically significant difference between controls and patients with *p* < 0.05/0.001, respectively.

## Data Availability

Because of the participant consent obtained as part of the recruitment process, it is not possible to make these data publicly available. The data resented in this study are available on request from the corresponding author.

## Supplementary Materials

The following supporting information can be downloaded at: https://www.mdpi.com/article/10.3390/metabo12070580/s1, Table S1. Selected characteristic of the study subgroups depending on BMI. Table S2. Selected characteristic of the study subgroups depending on PASI. Table S3. Selected characteristic of the study subgroups depending on the drug. Table S4. Correlations between the proteins and selected parameters in the study group before (a) and after (b) treatment. Table S5. Correlations between the proteins and selected parameters in the BMI subgroups (BMI 1—a, BMI 2—b, BMI 3—c) before and after treatment. Table S6. Correlations between the proteins and selected parameters in the PASI subgroups (PASI 1—a, PASI 2—b, PASI 3—c) before and after treatment. Table S7. Correlations between the proteins and selected parameters in the subgroups depending on the particular drug (ACY—a, MTX—b) before and after treatment.
