# Supplementary figures and images for: Potential Predictive Value of Serum Pentraxin 3 and Paraoxonase 1 for Cardiometabolic Disorders Development in Patients with Psoriasis—Preliminary Data
Source: Metabolites. 2022 Jun 22;12(7):580. doi: 10.3390/metabo12070580 (PMC9324570; doi:10.3390/metabo12070580)

## Slide 1
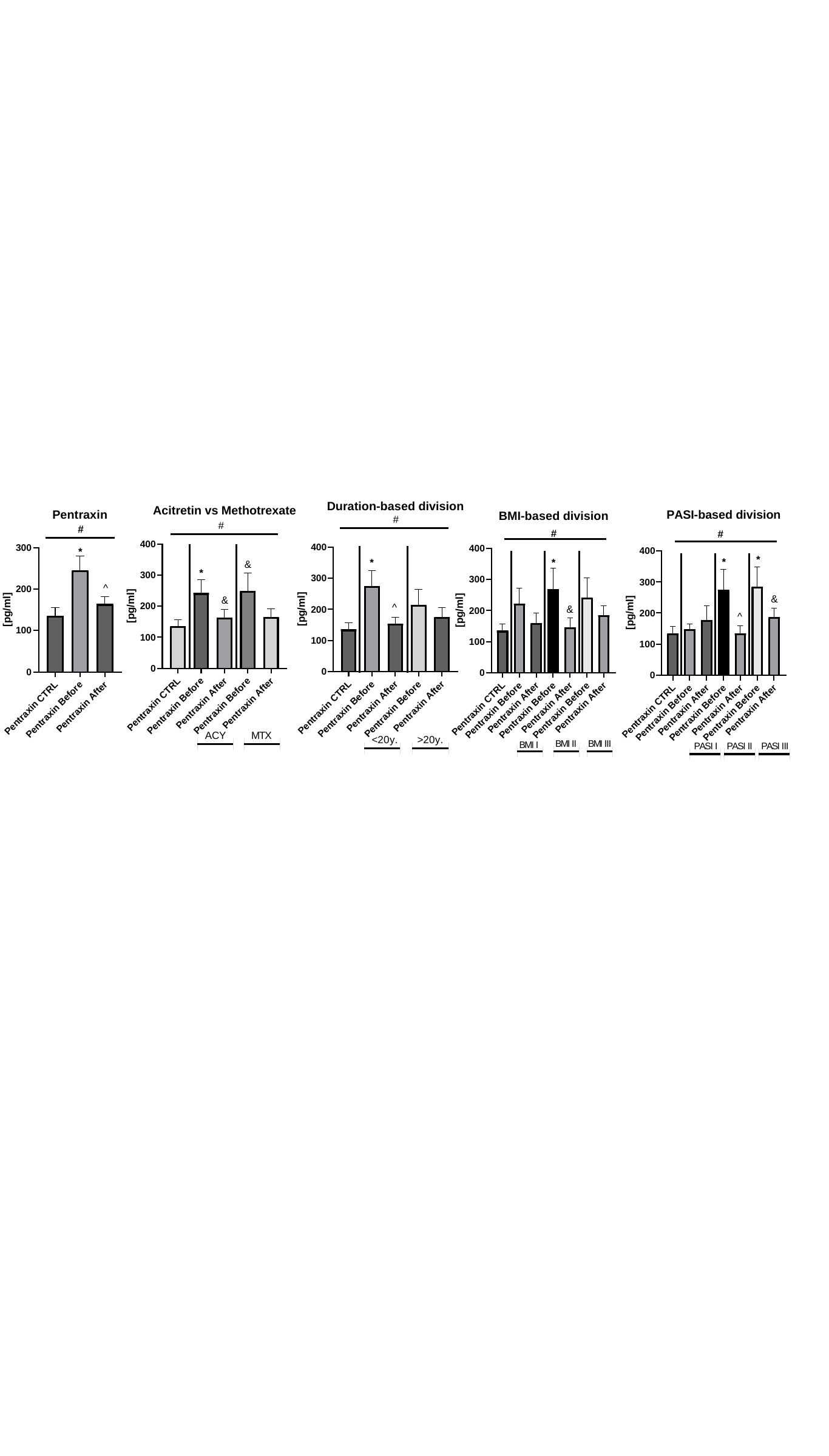

## Slide 2
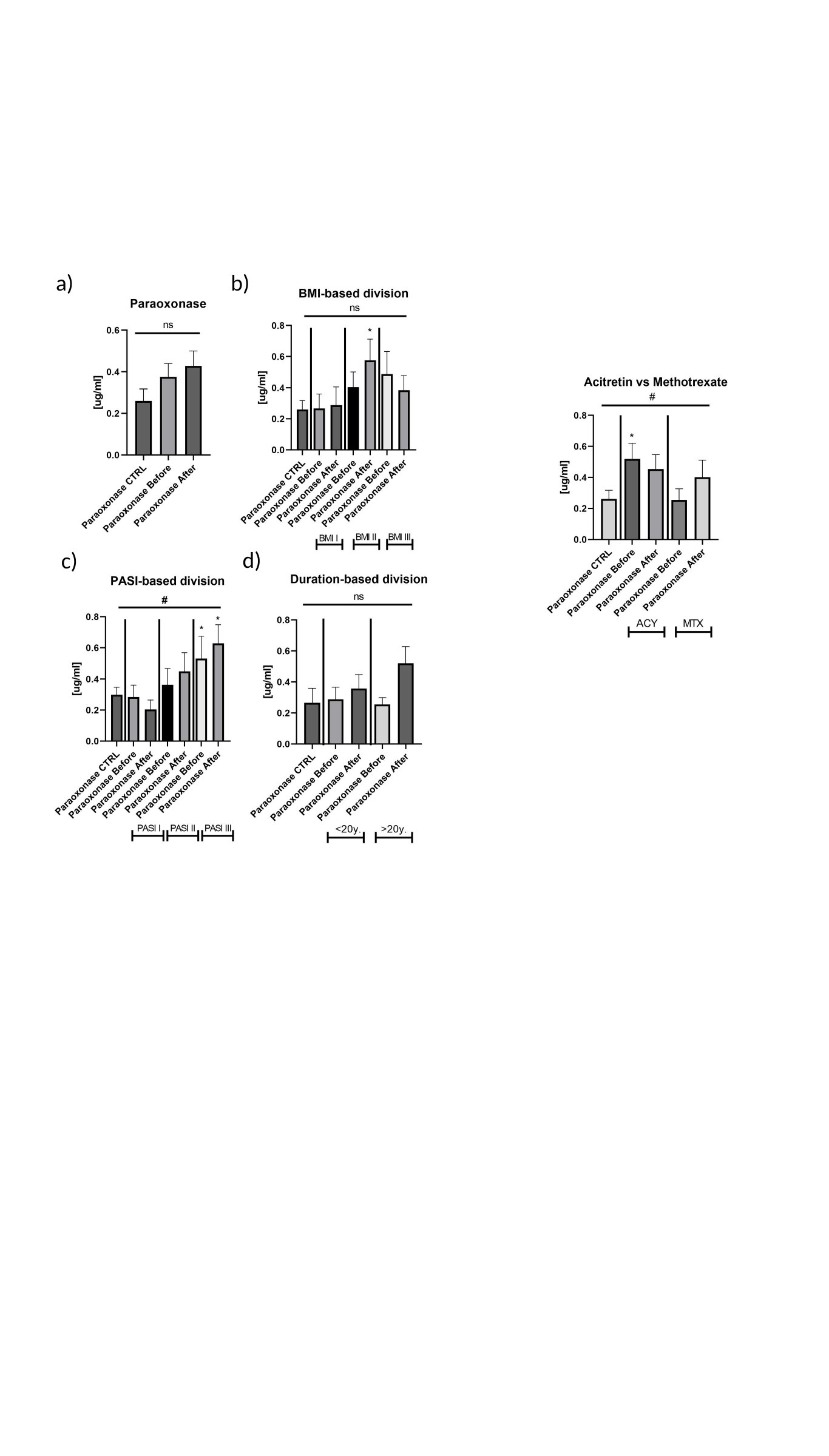

a)
b)
d)
c)

## Slide 3
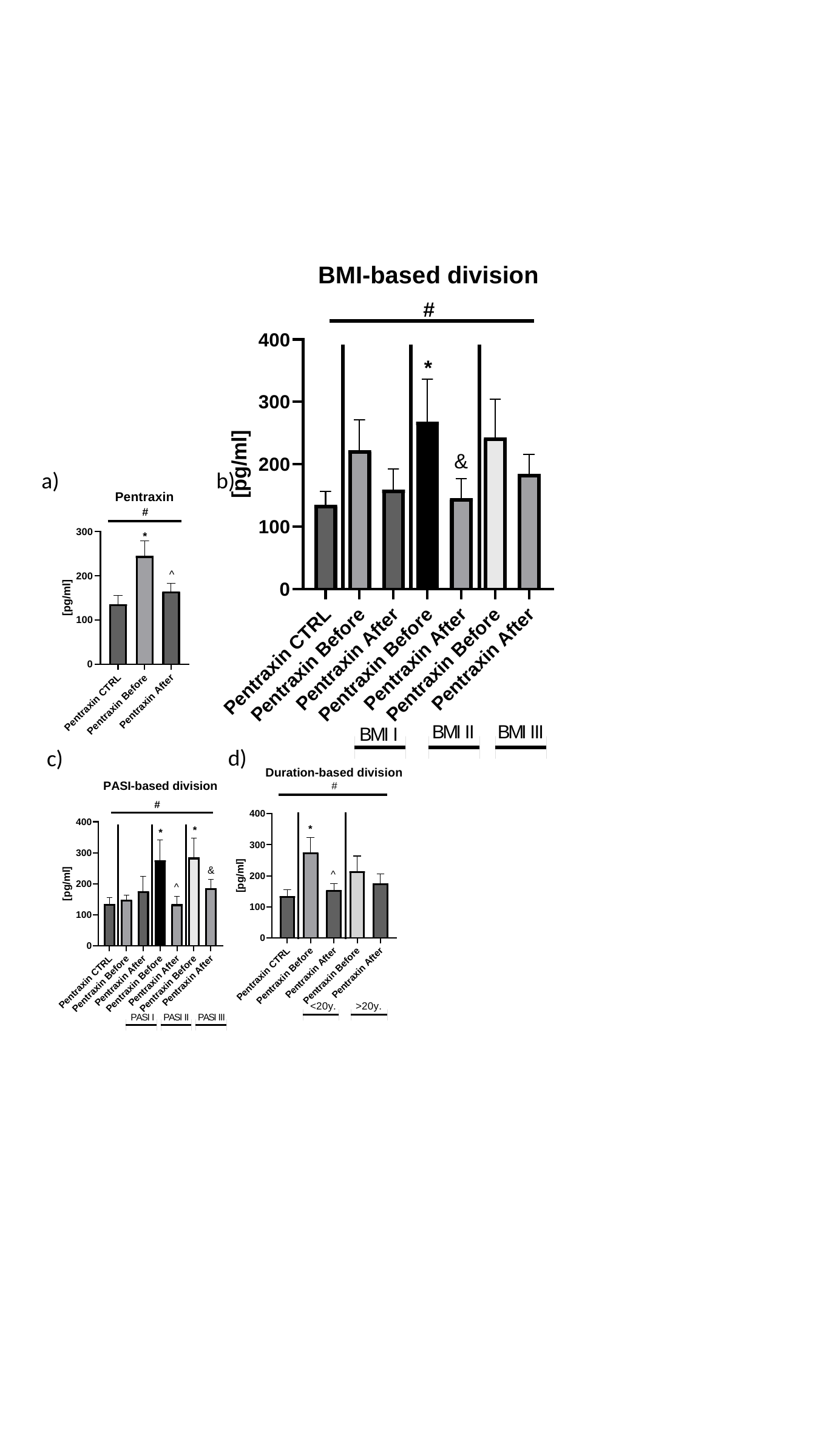

a)
b)
d)
c)

Supplement: Supplementary file 1 [file metabolites-12-00580-s001.zip › Wykresy_Glowne (1).pptx]
